# Communication Sparsity in Distributed Spiking Neural Network Simulations to Improve Scalability

**DOI:** 10.3389/fninf.2019.00019

**Published:** 2019-04-02

**Authors:** Carlos Fernandez-Musoles, Daniel Coca, Paul Richmond

**Affiliations:** ^1^Automatic Control and Systems Engineering, University of Sheffield, Sheffield, United Kingdom; ^2^Computer Science, University of Sheffield, Sheffield, United Kingdom

**Keywords:** Spiking Neural Networks, distributed simulation, hypergraph partitioning, dynamic sparse data exchange, HPC

## Abstract

In the last decade there has been a surge in the number of big science projects interested in achieving a comprehensive understanding of the functions of the brain, using Spiking Neuronal Network (SNN) simulations to aid discovery and experimentation. Such an approach increases the computational demands on SNN simulators: if natural scale brain-size simulations are to be realized, it is necessary to use parallel and distributed models of computing. Communication is recognized as the dominant part of distributed SNN simulations. As the number of computational nodes increases, the proportion of time the simulation spends in useful computing (computational efficiency) is reduced and therefore applies a limit to scalability. This work targets the three phases of communication to improve overall computational efficiency in distributed simulations: implicit synchronization, process handshake and data exchange. We introduce a connectivity-aware allocation of neurons to compute nodes by modeling the SNN as a *hypergraph*. Partitioning the hypergraph to reduce interprocess communication increases the sparsity of the communication graph. We propose dynamic sparse exchange as an improvement over simple point-to-point exchange on sparse communications. Results show a combined gain when using hypergraph-based allocation and dynamic sparse communication, increasing computational efficiency by up to 40.8 percentage points and reducing simulation time by up to 73%. The findings are applicable to other distributed complex system simulations in which communication is modeled as a graph network.

## 1. Introduction

### 1.1. Need for Distributed Computing

In recent years, there has been a growing scientific focus on computational neuroscience as a means to understand the brain and its functions, particularly at large, brain-size scale (Alivisatos et al., 2012; Koch, 2012; Markram, 2012; Amunts et al., 2016; Poo et al., 2016). Commonly accepted as the *third pillar of science* after theory and experimentation, simulation plays a central role in these projects. Thus, there is a need to run larger and more complex simulations.

Simulating brain-size models is computationally challenging due to the number and variety of elements involved and the high level of interconnectivity between them (Ananthanarayanan et al., 2009). The computing resources required at the brain scale far surpass the capabilities of personal computers today and will require compute power at the exascale (Markram, 2012). The slow down in the speed gains of individual cores due to physical limitations (Asanovic et al., 2006; Hasler and Marr, 2013; Marr et al., 2013) has made parallel and distributed computing the path to increased computational power. Although highly parallel devices such as GPUs have brought increased performance, to achieve the best computational power researchers resort to distributed computing, interconnecting computing nodes to escape the limitations of single devices.

This work focuses on distributed Spiking Neuronal Network (SNN) simulations and the challenges that arise from them, to make efficient use of available High Performance Computing.

Throughout the paper the term *process* will be used to denote an independent computation thread running exclusively on a physical core—and for our purposes, synonym of computing node. In the context of distributed communication, using the widely accepted standard MPI, a process is equivalent to an MPI rank.

### 1.2. Time Step-Driven SNN Simulations

Simulations of Spiking Neuronal Networks consist of discrete time-steps at which the membrane potential of the neurons involved is recalculated. When the potential reaches a certain threshold, neurons *fire*, producing a spike that is transmitted to connecting neurons. This spike in turn affects the membrane potential of receiving neurons. Thus, the simulation is divided in two phases: *computation phase*, at which neuron and synaptic models are updated based on partial differential equations; and *exchange phase*, where the spikes are propagated from firing neurons to post-synaptic targets.

In distributed systems, the exchange phase involves interprocess communication when pre- and post-synaptic neurons are hosted in different processes. In such cases, interprocess dependencies are introduced that force them to be *implicitly synchronized* (at the same step in the simulation) before starting *data exchange* (sending and receiving spiking information).

### 1.3. Bottleneck in Distributed Simulation

**Figure 1:**
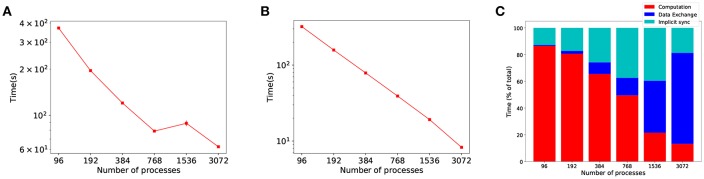
Evidence of the communication problem in large-scale distributed SNN simulations. The graphs show strong scaling results for a simulation with the same communication and computation time step, δ_*syn*_ (synaptic delay) and τ_*step*_ (time step) both set to the same value (0.1 ms), which is known to be the worse case scenario in communication. **(A)** Simulation time as the number of processes is increased. **(B)** The time the simulation spends in computing. **(C)** Proportion of time the simulation spent in each phase, with communication (data exchange and implicit synchronization) becoming the dominant part.

Past approaches to parallel and distributed neuronal simulators have focused their efforts on load balancing (Gewaltig and Diesmann, 2007; Kunkel et al., 2014), and not on the communication overhead of increased parallelism. However, in large scale SNN distributed simulations, propagation of spikes between processes has been suggested as the bottleneck for scalability (Brette and Goodman, 2012; Zenke and Gerstner, 2014). To demonstrate this, Figure 1 shows a strong scaling experiment of the Cortical Microcircuit (Potjans and Diesmann, 2014) distributed simulation with frequent communication (at every time step) and random allocation of workload, i.e. neurons to processes^1^. Whilst computation shows near-perfect scaling (Figure 1B), the proportion of time the simulation spends in communication scales poorly (both shades of blue in Figure 1C), becoming the dominant part as the number of processes is increased.

We define computational efficiency as the proportion of time a distributed simulation spends in the computation phase. Figure 1A shows overall simulation time stagnation in strong scaling experiments, as a result of poor communication scalability. Even though the computation part of the simulation scales well (Figure 1B), the benefits of increased parallelism are limited by communication—as shown by the shift in time spent in communication and computation in Figure 1C.

### 1.4. Key Contributions

**Figure 2:**
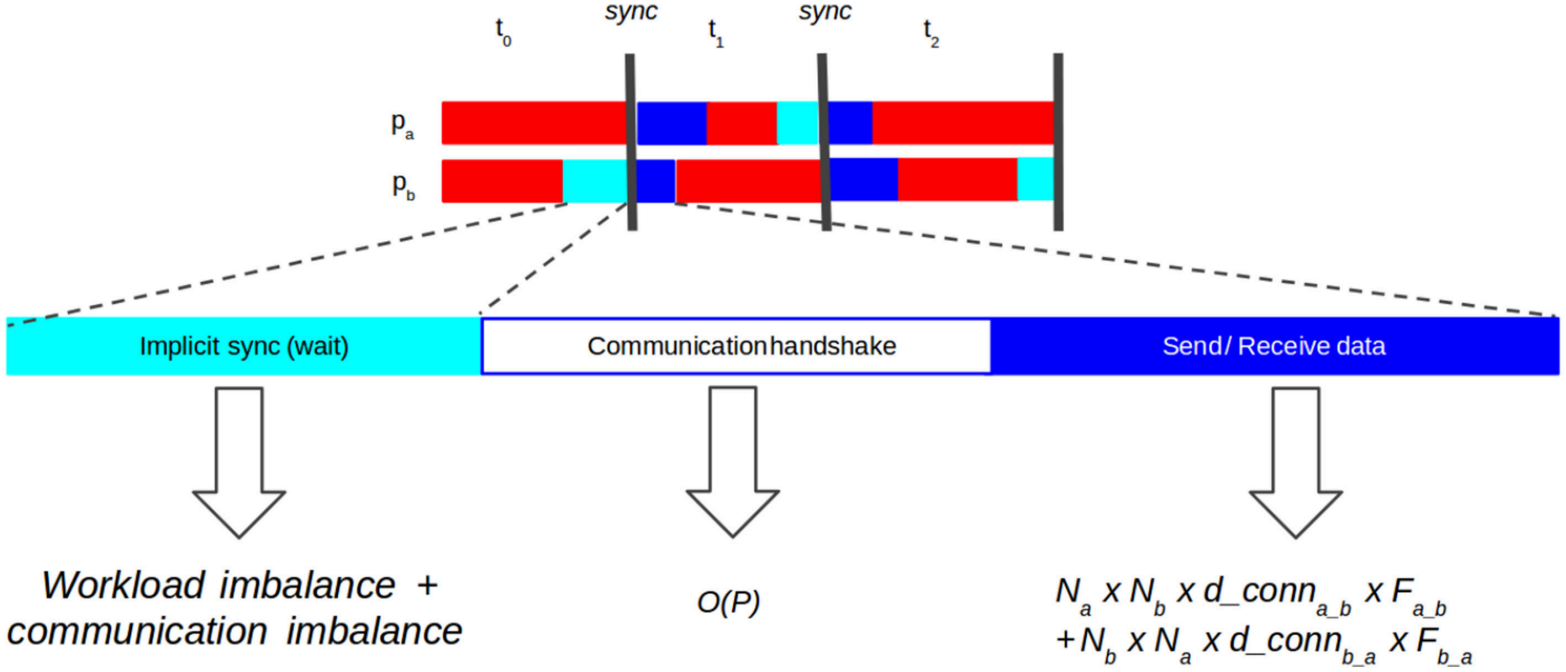
Diagram showing synchronization phases in point-to-point (P2P) communication. Implicit synchronization (light blue): processes waiting for each other to start communication. Communication handshake (white): explicit notification to other processes of the intention to send data (receiver processes must prepare buffers). Send-receive data (dark blue): processes send spiking data to each other. The cost of the send-receive data is determined by the number of neurons on two communicating processes (*N*_*a*_ and *N*_*b*_), how densely connected those populations are (*d*_*conn*_*a*_*b*_ and *d*_*conn*_*b*_*a*_) and the frequency at which those populations communicate (*F*_*a*_*b*__ and *F*_*b*_*a*__).

State-of-the-art SNN distributed simulators have demonstrated the effectiveness of the use of point-to-point (P2P) communications (Hammarlund and Ekeberg, 1998; Kumar et al., 2010; Minkovich et al., 2014; Jordan et al., 2018). Using P2P introduces a necessary handshake protocol between processes to know which processes each one has to listen to. Thus, each communication stage consists of three phases (Figure 2):

1. Implicit synchronization, in which processes must wait until every other process is ready for communication. Computation and communication imbalance contribute to this phase.
2. Communication handshake (informing target processes of the intent to communicate). In naive P2P communication this operation has O(P) complexity (Hoefler et al., 2010b), with *P* number of processes, a problem for scalability.
3. Data exchange (actual send and receive of spiking data). The cost of the send-receive data is determined by the number of neurons hosted on two communicating processes, their interconnection density and the frequency of communication, i.e., the firing rate.

The key contribution of this paper is to exploit network communication sparsity in SNN graphs to improve the three phases of point-to-point communication in distributed SNN simulations. Two proposed strategies contribute to those phases: connectivity-aware neuron allocation to processes with *hypergraph partitioning* improves workload balance (implicit synchronization), increases communication sparsity by reducing the number of neighboring processes (communication handshake) and decreases the density of communication between process neighbors (send-receive data); *dynamic sparse data exchange* as an interprocess communication pattern improves communication balance (implicit synchronization) and reduces data exchange by overlapping communication handshake and send-receive data. Furthermore, there is a synergy between the effects of connectivity-aware allocation and sparse communication to increase computational efficiency in distributed SNN simulations.

### 1.5. Previous Work

#### 1.5.1. Optimization of Spike Propagation

In a continuous SNN simulation run in distributed processes, after updating the states of neurons and synapses, each process needs to broadcast spike data so target postsynaptic neurons are notified of the activity of presynaptic neurons. This synchronization event occurs at defined time intervals, with communication overhead at every step. One method of reducing the overhead is to pack event-messages together by using the intrinsic synaptic delay property of the neuronal connection (Morrison et al., 2005; Gewaltig and Diesmann, 2007). The global minimum synaptic delay (smallest synaptic delay in the simulation) is used to set the frequency at which processes need to communicate. Hammarlund and Ekeberg (1998) take this idea further and use a different frequency of communication per process pair, defined by the minimum synaptic delay in synapses across those processes, with a reduced overall communication overhead.

Most distributed SNN simulators use the Address Event Representation (AER) to compress the spike data exchanged between parallel processes. In software simulators, the AER approach by Morrison et al. (2005) is commonly used, where synapse objects are stored where the post-synaptic neuron is placed. Thus, processes containing firing neurons need only send the neuron ID and time stamp information to other processes. *HRLsim* (Minkovich et al., 2014) dynamically switches to an alternative bit representation (Boahen, 2000) when the activity of the network is high. The bit representation describes the state (fired or not fired) of an ensemble of 32 neurons with a bit in a 4 bytes integer, allowing to represent the entire network more efficiently. Nageswaran et al. (2009) targets the time stamp data to reduce the size of event messages. Instead of sending neuron id-timing pairs, they employ timing tables at which processes attach spike data, removing the need to include time data. Similarly, Morrison et al. (2005) avoid having to send individual spiking timestamp by adding markers to the spike buffers (one per time interval between communication events), thus receiving processes are able to determine when the remote spike occurred.

After compressing and packing spiking data, parallel simulators must broadcast the messages to any and all processes that require it. Main software simulators (Carnevale and Hines, 2006; Gewaltig and Diesmann, 2007; Hoang et al., 2013; Zenke and Gerstner, 2014) take the simple approach of sending every message to all other processes (*all gather*). Although this design scales poorly, it is easy to implement and for lower processes counts the hardware optimizations made for such a collective function call may hide this cost. To support the *all gather* approach, Lytton et al. (2016) suggest that it may perform better than a discerning point-to-point, provided the number of processes involved is significantly lower than the average number of connections per neuron. In this situations, the probability that any neuron has post-synaptic targets in any other process is high, hence most spiking messages need to be sent to all other processes. In large scale simulations, however, with the order of thousands of processes, this may not be the case, as the average neuron connectivity will quickly be outnumbered by the number of processes, making all gather communication inefficient. The point at which average neuron connectivity outweighs the number of processes is dependent on the model in question; for human brain models it can reach tens of thousands, but for specific microcircuits it is significantly below that, such as the cortical microcircuit (3.8 k, Potjans and Diesmann, 2014) and the multi-area macaque visual cortex (5.8 k, Schmidt et al., 2018). This relationship between average neuron connectivity and number of processes is behind the performance demonstrated by Kumar et al. (2010) in their extension to *NEURON*, which showed higher gains in communication efficiency in low connectivity density models.

Previous works (Migliore et al., 2006; Jordan et al., 2018) acknowledge that the communication overhead using *all gather* becomes a problem for scalable parallel simulations and better broadcasting efficiency is needed. *SPLIT* (Hammarlund and Ekeberg, 1998) and *HRLSim* (Minkovich et al., 2014) both implement point-to-point communication with reported gains over *all gather*. Jordan et al. (2018) propose using *all to all* as an alternative collective communication in large scale *NEST* parallel simulations. Although they are more interested on reducing the memory footprint of communication at large scale, this collective method minimizes the redundancy of data sent across the simulation, with each process sending unique data to all other processes.

In large scale simulations performed to date, communication overhead is dealt with in several ways for each case. Kunkel et al. (2014) and Jordan et al. (2018) rely on message packing to reduce the communication overhead problem (communicate only every 15 time steps). In their custom cortical simulator Ananthanarayanan and Modha (2007a,b); Ananthanarayanan et al. (2009) use a point to point communication pattern to reduce communication volume. Their work includes strong scaling experiments showing that as the number of processors is increased, the simulation becomes communication bound. Other large scale simulations do not explicitly optimize communication as they do not study scalability (Izhikevich and Edelman, 2008; Eliasmith et al., 2012).

#### 1.5.2. Allocation of Neurons to Processes

Most SNN parallel simulators use round-robin allocation of neurons to computing nodes (Migliore et al., 2006; Gewaltig and Diesmann, 2007; Pecevski et al., 2009; Yavuz et al., 2016). This can be seen as a simple form of static load balancing (Kunkel et al., 2014). Although authors have proposed models to account for heterogeneous workload balancing (Hoang et al., 2013), very little work has focused on the impact that neuron allocation has over simulation communication (i.e., spike propagation). *HRLSim* (Minkovich et al., 2014) suggests assigning neurons based on how tightly connected they are but without implementation details. Urgese et al. (2016) present an improvement to the default division of workload policy *PACMAN* in *SpiNNaker* (Galluppi et al., 2012). They use spectral clustering to group neurons into sub-populations, where tightly connected groups are kept in the same computational node (process). A similar approach, but based on graph partitioning is employed in the complex system simulator *InsilicoSim* (Heien et al., 2010), demonstrating the potential benefits of graph partitioning to distribute computation.

#### 1.5.3. Communication in Large Scale Distributed Applications

Thakur et al. (2010) argue that at the peta and exascale computing, current communication patterns in MPI (the standard library for distributed computing) struggle to scale. The main two challenges are memory footprint of irregular collective operations and poor scaling of collective communications (Balaji et al., 2009). The former is due to the requirement to define buffers the size of the number of processes as function arguments in every irregular collective call. The latter is a consequence of the nature of all to all communications, each process sending data to all others, with little opportunity for optimization—although work has been done to improve performance on specific hardware (Mamidala et al., 2008; Adachi et al., 2012). Since brain scale simulations will require large scale distributed architectures, SNN simulations will have to deal with those issues too.

Neighborhood collective methods introduced in MPI 3.0 (Hoefler and Träff, 2009) are an improvement over normal collective methods but they require a predefined communication graph to know process neighborhood. This static exchange requirement makes it hardly applicable for SNN simulation where infrequent communication and plasticity are involved. Furthermore, neighbor messaging is only advantageous if the average size of a process neighborhood (i.e., the number of neighbors it communicates with) is significantly lower than the total number of processes. Due to their high level of interconnectivity this is unlikely to be the case in biologically plausible SNNs.

In other complex system simulations (such as molecular interactions, fluid dynamics, etc.), computation is divided based on position and communication tends to happen primarily (or exclusively) in the overlapping or adjacent locations. Communication in parallel SNN simulations is very different since neurons can communicate (i.e., spike) with potentially any other neuron in the model, irrespective of their location. The format of the communication is also unique, as it takes the form of low frequency, discrete messages (spikes) from one neuron to all of its post-synaptic targets. These two facts make communication in SNNs very dynamic (target neighbors change across the simulation) and sparse (target neighbors at each communication step are a subset of the total). Hoefler et al. (2010b) proposes *Dynamic Sparse Data Exchange* (*DSDE*) algorithms for scalable sparse communication in large neighborhoods with good scalability. This work explores the benefits of *DSDE* as a communication pattern in SNN simulations.

### 1.6. Summary of the Paper

The remainder of the paper is organized as follows. Section 2 details the proposed methods to allocate neurons to processes using hypergraph partitioning and dynamic sparse point-to-point communications. Section 2.3 outlines the details of the experiment setup, including the neuronal model developed for this work. Sections 3 and 4 present and discuss the results obtained from scalability analysis on the proposed approaches, comparing them with baseline implementations.

## 2. Materials and Methods

**Figure 3:**
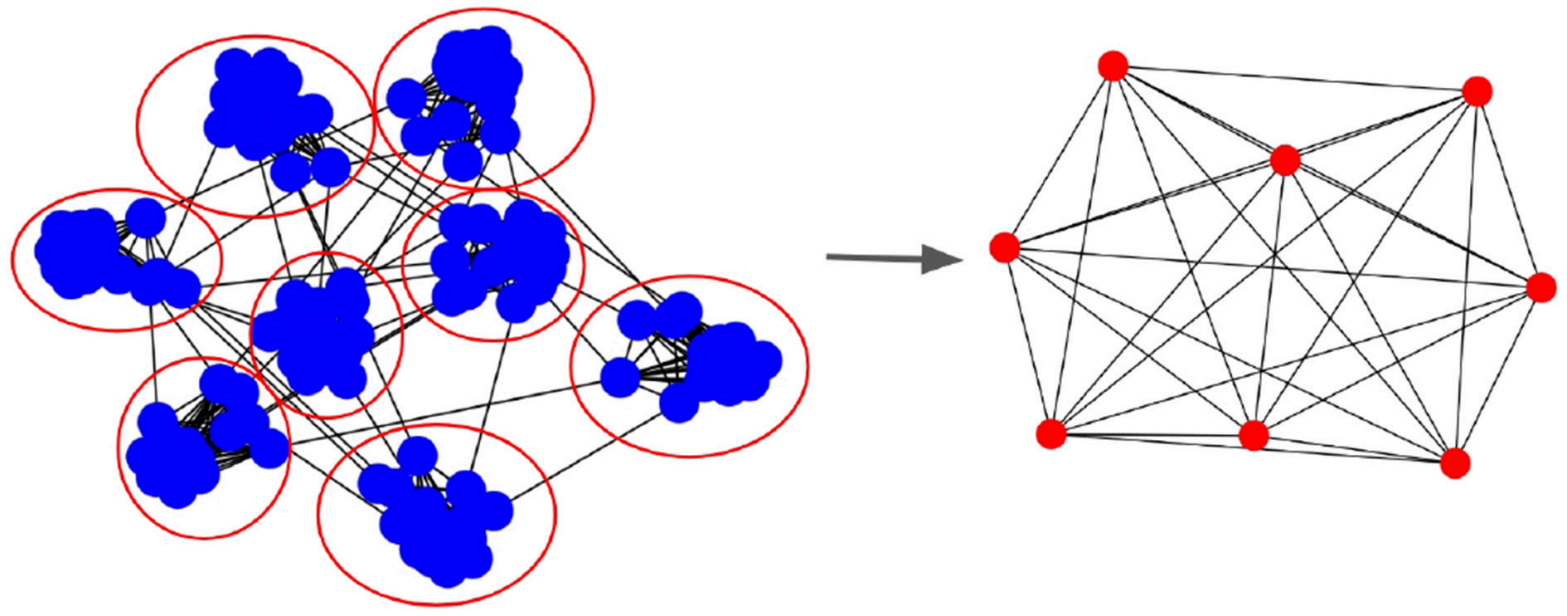
Process Communication Graph (PCG) that represents parallel SNN simulation communication. **(Left)** The graph with blue nodes represents the SNN synaptic connectivity and the red circles are processes to which the neurons are mapped. **(Right)** Resulting process graph in which edges represent processes that need to synchronize during simulation, i.e., have an inter-process synaptic connection between themselves. The PCG describes the process neighborhood for each computing node.

Communication in parallel SNN happens due to pre- and post-synaptic neurons being hosted by different processes. Figure 3 shows the Process Communication Graph (PCG) resulting from a mapping of neurons to 8 processes. In the PCG, edges are shown only if there are *any* synapses between neurons of both populations. As a consequence, during simulation, each process has to synchronize (i.e., receive information) with every neighboring process it has in the PCG.

This work tackles the communication overhead issues that limit distributed scalability in large scale SNN simulations. Our goal is to increase computational efficiency by reducing the time simulations spend on communication between processes. We focus on reducing the overhead of communication by: (1) using a connectivity-aware allocation of neurons to compute nodes; and (2) employing scalable sparse parallel communication patterns. These two strategies are complementary and address the sparsity of communication in the PCG.

### 2.1. Hypergraph to Represent Connectivity in a SNN

Since communication in distributed SNN simulations only happens when there is interprocess connectivity in the PCG, minimizing this connectivity directly reduces the communication requirements. The topology of biological plausible complex neuronal networks is found to show presence of clusters, where local connectivity is expected to outnumber remote connectivity (Bullmore and Sporns, 2009). Hence, there is an opportunity to optimize communication by considering this clustering when assigning neurons to compute nodes.

We propose to model the SNN as a hypergraph. A hypergraph has been shown to successfully model total communication in parallel applications (Devine et al., 2005; Deveci et al., 2015). Formally, a hypergraph *H* = (*V, E*) consists of a set of vertices *V* and a set of hyperedges *E*, where each hyperedge is a subset of *V* that defines the connectivity pattern. Note that a graph is a specific case of a hypergraph in which the cardinality (size) of each hyperedge is equal to 2—hence each edge connects a pair of vertices. In hypergraphs, however, hyperedges can have any cardinality, i.e., one hyperedge connects multiple vertices.

**Figure 4:**
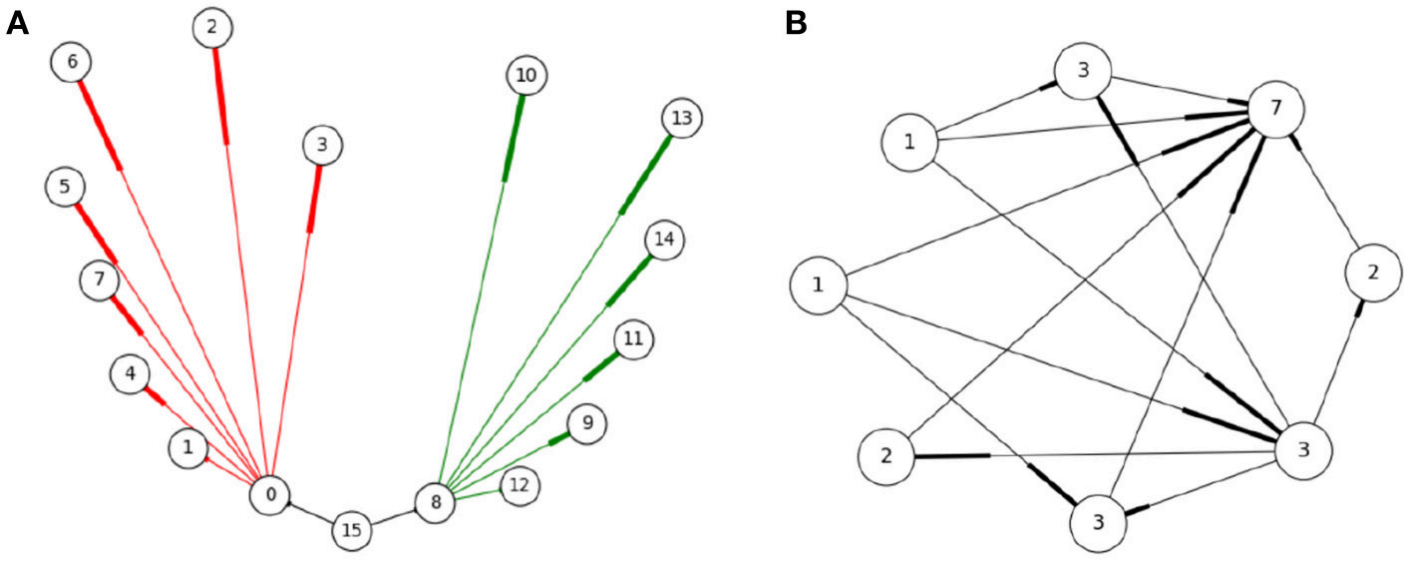
Representing the SNN as a hypergraph **(A)**: Three hyperedges (black, red, and green) shown, where a hyperedge includes a pre-synaptic neuron and all its post-synaptic neurons (the numbers describe neuron IDs). This representation corresponds to the manner neurons communicate, i.e., when a neuron fires all its post-synaptic targets need to be notified. **(B)** The hypergraph nodes are weighed by the number of dendritic inputs to a neuron as an estimate of the workload located at each process. In this example, edges represent synaptic connections between neurons (nodes), with the thicker end indicating direction. The number on the node represents the weight associated to each neuron, equal to the sum of its input plus one.

A SNN can be thought of as graphs with neurons as nodes and synaptic connections as edges. To better model the cost of communication, instead of using edges (one to one connectivity) we use hyperedges. Each hyperedge contains a pre-synaptic neuron and all of its post-synaptic targets—see Figure 4A. This captures the all-or-nothing communication behavior of neurons, where when a neuron spikes, a message is sent to all its post-synaptic targets (and not a subset of targets). Thus, a hyperedge adequately models the unit of communication in a SNN. To model workload, hypergraph nodes are weighed by the number of dendritic inputs —Figure 4B. The number of inputs is a good estimate of the workload associated with hosting a neuron; synaptic objects handling dendritic input computation are located on the post-synaptic side, therefore more dendritic input requires more computation during the synaptic update phase.

#### 2.1.1. Hypergraph Partitioning for Neuron Allocation

When neurons within the same hyperedge are assigned to different nodes of the PCG, communication between them is required during simulation. Note that interprocess communication is therefore not required if there are no hyperedges spanning more than one process. Hence, the allocation of neurons to processes can be formulated as an optimization problem where the goal is to reduce the number of hyperedges cut between processes. Multilevel hypergraph partitioning, generally understood to produce better partitions faster than other alternatives (Hendrickson and Leland, 1995), is used. Multilevel partitioning algorithms are a family of procedures that divide the input graph in three distinct phases: coarsening or simplification of the graph by merging nodes together; partitioning, where the simplified graph is divided into portions; and expansion, where the graph is uncoarsened to its original form and the partitions are refined. This work makes use of the *Zoltan* library (Boman et al., 2012), with the agglomerative inner product matching coarsening method (Catalyurek et al., 2009), and FM refinement method (Fiduccia and Mattheyses, 1982). To better represent the communication costs, each hyperedge cut is weighed based on the number of participant parts minus one. Formally, the total cost of a partition scheme is $\overset{\left|E\right|}{\underset{i=0}{\sum }}P\left(e_{i}\right)$, where *e*_*i*_ = {*p*_1_, *p*_2_…, *p*_*n*_} represents the set of partitions that contain any node in hyperedge *e*_*i*_, and *E* is the set of all hyperedges. The cost *P*(*e*) of hyperedge *e* is defined as *P*(*e*) = |*e*|−1.

To avoid trivial solutions that minimize the hyperedge cut (such as assigning all vertices to one partition) partitioning algorithms maintain load balancing by only allowing solutions that have a total imbalance factor that is below a specified value. The total imbalance is calculated dividing the maximum imbalanced partition in the scheme by the average imbalance across partitions. Formally:

$$\frac{\max_{p\in P}\left(L\left(p\right)\right)}{\left(\sum_{i=0}^{\left|P\right|}L\left(p_{i}\right)\right)/|P|}$$

where *P* is the set of partitions and *L*(*p*) is the load cost for partition *p* defined as the sum of the weights of all its nodes, $L\left(p\right)=\overset{N}{\underset{i=0}{\sum }}W\left(n_{i}\right)$ where *N* is the number of nodes in partition *p* and *n*∈*p*. The total imbalance must be lower or equal than an arbitrary tolerance value (in our experiments, 1.001). The workload of each node in the hypergraph (neuron) is estimated by the number of incoming post-synaptic connections, and using that as the weight of the node (see Figure 4). The computation in the simulation is dominated by synaptic updates, based on experimental validation. Post-synaptic objects live in the same partition local to the post-synaptic neuron, hence those partitions with more incoming synapses will have more computation to perform during updates.

### 2.2. Scalable Dynamic Sparse Communication Patterns

Communication in SNN simulations falls into the category of *census*, a common parallel programming function in which a process receives a piece of data from each of all other processes. Each process knows to whom it needs to send data (any process hosting post-synaptic targets of spiking local neurons), but has no information as from whom it is going to receive data (non-local firing neurons). Personalized census or personalized exchange (*PEX*) is the most basic implementation of census (Hoefler et al., 2010b) in which communication occurs in two steps: (1) interprocess handshake and (2) send and receive data. During handshake, processes inform their targets that they will be sending data to them. In the second phase, each process post data and listens to messages only from those processes.

**Figure 5:**
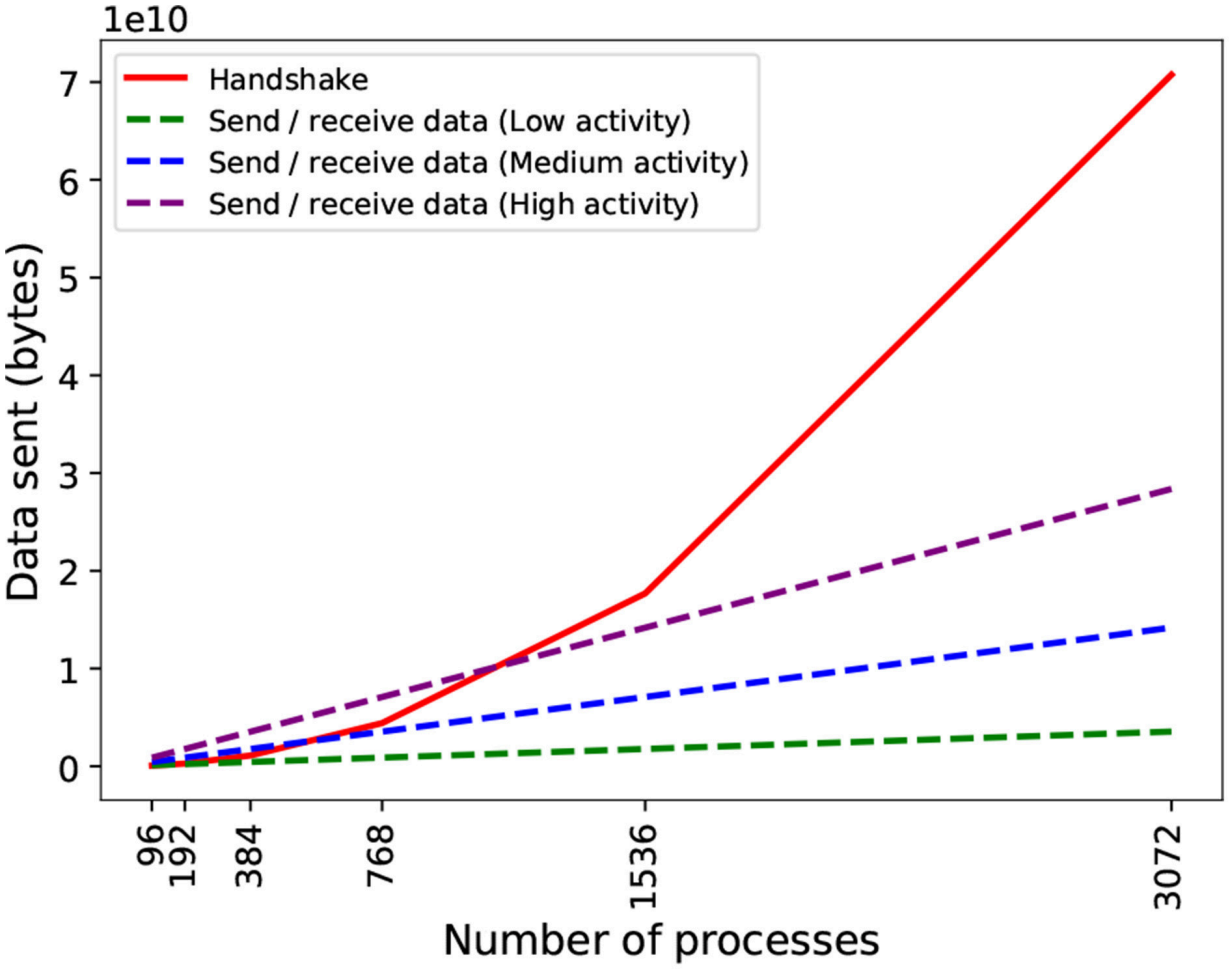
Theoretical data volume exchanged in the two phases of communication by *PEX* on an artificial network with constant spiking activity (20 spikes/s for low activity, 80 spikes/s for medium activity, and 160 spikes/s for high activity). During spiking data exchange, processes send data to each other in two phases: handshake (coordinating intention to send information) in solid red line; and send-receive spiking data, in dashed lines. The send-receive exchange volume is dependent on the activity density (spiking average) per process. The communication profile of the simulation and the number of processes used determines which phase is dominant.

With respect to scalability, there are two issues with *PEX*: on the one hand, due to the dependency between phases, they occur sequentially, i.e., each process must wait until it has completed the handshake before sending data. This adds waiting time that is dependent on the total number of processes (messages will take longer to propagate in larger topologies). On the other hand, during handshake, each process sends metadata to all others, even if they do not need to send spiking data to them (they still need to inform others that they will not be receiving data). This causes an overhead of metadata with a quadratic growth with the number of processes (Figure 5) and quickly becomes the dominant part of the exchange.

**Figure 6:**
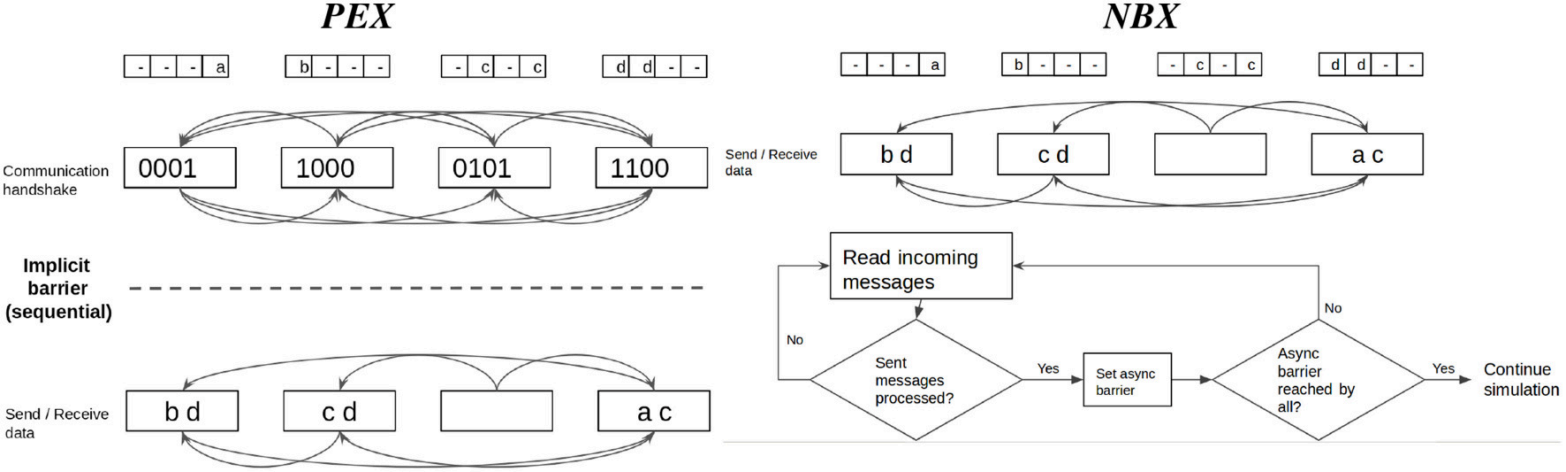
Point-to-point communication strategies compared. Personalized Exchange (*PEX*) involves an explicit handshake to coordinate communication intention amongst processes before exchanging data. First, processes share metadata in an all to all manner; in a second round, processes send data only to those that need to receive spiking data. Neighbor Exchange (*NBX*) overlaps both phases with the use of asynchronous barrier, which allows processes to send and receive spiking data while waiting for notification that other processes have completed sending data, minimizing synchronization time.

Neighborhood Exchange (*NBX*) belongs to the class of *Dynamic Sparse Data Exchange* algorithms proposed by Hoefler et al. (2010b). It targets the overhead (both waiting time and metadata exchange) caused by the handshake phase of communication by overlapping it with the send-receive data phase—see Figure 6). For sparse communication, Hoefler et al. (2010b) demonstrate that *NBX* performs better than *PEX*.

Previous attempts to optimize data exchange in SNN simulators have been limited to implemented *PEX*-like point-to-point communication (Hammarlund and Ekeberg, 1998; Ananthanarayanan and Modha, 2007a; Kumar et al., 2010; Minkovich et al., 2014; Jordan et al., 2018). To the best of our knowledge, no SNN simulation has considered using *DSDE* algorithms. This work compares the performance of *PEX* and *NBX* and their impact to communication performance.

#### 2.2.1. Details on the Implementation of *PEX* and *NBX*

Both algorithms require each process to maintain a lookup table of target processes each local neuron connects to (populated before starting the simulation). When a process is in the communication stage, it matches the local spiking neurons to the lookup table to generate the data to be sent to each process. PEX and NBX differ in the way they distribute this data. Both *PEX* and *NBX* follow the implementations described by Hoefler et al. (2010b).

For the comparisons, *PEX* is implemented with an all to all call for the handshake, which informs other processes of which processes to listen to. An asymmetric all to all follows, where each process may send different amounts of data, to send-receive data (which is preferred to individual send and receive postings per process as it incurs in less call overheads).

*NBX* is implemented as depicted in Figure 6. First, each process sends data asynchronously (non-blocking) to all its targets. Whilst the messages are being delivered, processes probe for incoming messages. Once a sender process is notified that its messages have been received, it places an asynchronous barrier and continues to probe for incoming messages until all other processes have reached the barrier (with an MPI_Ibarrier call), signaling the end of the communication phase. The use of an asynchronous barrier removes the need for an explicit handshake, effectively allowing the data exchange to start without explicit knowledge from the receiver.

### 2.3. Experimental Design

#### 2.3.1. Cortical Microcircuit SNN Model

For the benchmark experiments, the model used is based on the Cortical Microcircuit (*CM*) described by Potjans and Diesmann (2014), scaled to 77k neurons and 150M synaptic connections. The *CM* model is representative of microcircuit-type models in which a SNN is constructed by layers of neurons and connectivity probabilities within and between layers. As such, findings on the CM model can be applicable to other microcircuit models and similarly structured biologically plausible networks.

The size of the model is sufficient to display the limitations of scalability in distributed simulations due to communication overhead scalability —see Figure 1. This is a consequence of the high frequency of synchronization required in the simulation: message packing optimization is not allowed because the model contains random synaptic delays, forcing processes to synchronize at each time step.

To ensure network activity across the simulation, uniform constant current is injected to all neurons (0.95 mA), with resting potential (−45 mV) and spiking threshold (−50 mV). This form of input differs from Potjans and Diesmann (2014) Poisson spike trains but results in an equivalent global average activity of 5–7 spikes/s. A constant input is more adequate for our study of communication in distributed simulations, as it tends to spread the activity across the network. This prevents from skewed communication (in which a subset of processes do most of the sending whilst others only receive), that could interfere with interpreting the results from allocation and communication algorithms. Biological realism in activity patterns is therefore not required.

#### 2.3.2. Macaque Visual Cortex Multi-Scale Model

To understand how the hypergraph partitioning allocation and the *NBX* communication scale to larger models, we implement the multi-scale model of the macaque visual cortex (*MVC*) described by Schmidt et al. (2018). The model bridges the gap between local circuit models (such as the *CM*) and large-scale abstractions to represent one hemisphere of the macaque visual cortex. It is comprised of 32 areas, each one constructed as a *CM* and connected to others with fixed probability, i.e., a cell on one area is connected to cells in another area with a probability that is fixed for all cells between said two layers. The nature of the model is modular (i.e. divided in areas) which makes it a suitable candidate for smart partitioning.

The model is implemented at 16% of the original scale, with 660 k neurons and approximately 620 million synapses, with the same connectivity probability. Neurons share the same parameters as the ones detailed in the original model (Potjans and Diesmann, 2014). As with the *CM* model, a constant current of 0.38 mA is introduced to all neurons. To produce an average activity of 20–23 spikes/s, the weight multiplier of inhibitory connections is set to −15 (instead of −4).

#### 2.3.3. Implementation of the Models

Both models are implemented in C++, where different neuron allocation and communication strategies are evaluated. The main features of the implementation are:

- Time-step driven simulation.
- Single neuron (leak integrate and fire) and synaptic models (exponential).
- Distributed across computing nodes (processes). Within a process, computation is performed sequentially (computation performance is out of the scope of this work).
- MPI library used for interprocess communication.

These features are built to be representative of the wider class of SNN simulators, with functionally equivalent output (network activity) for the same model execution. Thus, the findings on this work could inform development in other time-step, distributed SNN simulators (such as *NEST, NEURON, Arbor* Klijn et al., 2017).

Profiling timing is done internally by wrapping specific functions with MPI_Wtime calls. We are mainly interested in measuring time spent in computation, in implicit synchronization and in data exchange, as well as how these impact overall performance.

- Computation: includes the sequential computation of neuron and synapse state updates, as well as effecting the received spiking information from other processes to local neurons.
- Implicit synchronization: to quantify load balance, a global artificial barrier is introduced at the end of the computation cycle to measure waiting time on each process^2^
- Data exchange: measurement of the time it takes for the selected communication pattern to initiate and complete spike propagation.
- Simulation time: overall global time to complete the simulation, including computation, implicit synchronization, and data exchange.

The code for the implementation of the models, as well as the allocation and communication algorithms have been made available and can be accessed at: https://github.com/cfmusoles/distributed_sim.git.

#### 2.3.4. Hardware Architecture

Simulations of the CM and the MVC model are run on the ARCHER Cray XC30 MPP supercomputer, with 4,920 nodes, each with two 12 cores Intel Ivy bridge series processors (for a total of 24 processors) and up to 128 GB RAM available. Computing nodes are connected via fast low latency Cray Aries interconnect links^3^.

ARCHER allocates exclusive computing nodes (cores and memory), however, as a cluster computer, network related resources are potentially shared. There are two types of noise that can affect benchmarking results: external application traffic contention and distance on the allocated computing nodes. Traffic noise is minimized by running each iteration (same seed) of an experiment multiple times (with the same node allocation) and selecting the fastest sample. Node distance variability (communication between any two nodes depends on how close they are in the topology) is smoothed by running each set of experiments multiple times with different node allocations. Furthermore, when comparing strategies, all candidates within an experiment set run with the same node allocation.

## 3. Results

The first series of strong scaling experiments (size of the model is fixed whilst increasing the number of processes) are run to understand the impact of the novel neuron allocation strategy and the application of *DSDE* communication patterns in SNN simulation. The experiments simulate 750 ms of the *CM* model at 0.1 ms time step^4^. The process scales used are: 96, 192, 384, 768, 1,536, and 3,072 processes.

### 3.1. Neuron Allocation via Hypergraph Partitioning

The baseline neuron allocation strategy *Random Balanced* is a variation of random allocation that takes the number of post-synaptic connections into account to keep processes balanced. *Random Balance* performance is compared to *Hypergraph*, our allocation strategy that uses hypergraph partitioning to minimize the total interconnectivity between nodes in the Process Communication Graph. Both strategies use *PEX* to communicate spiking data.

**Figure 7:**
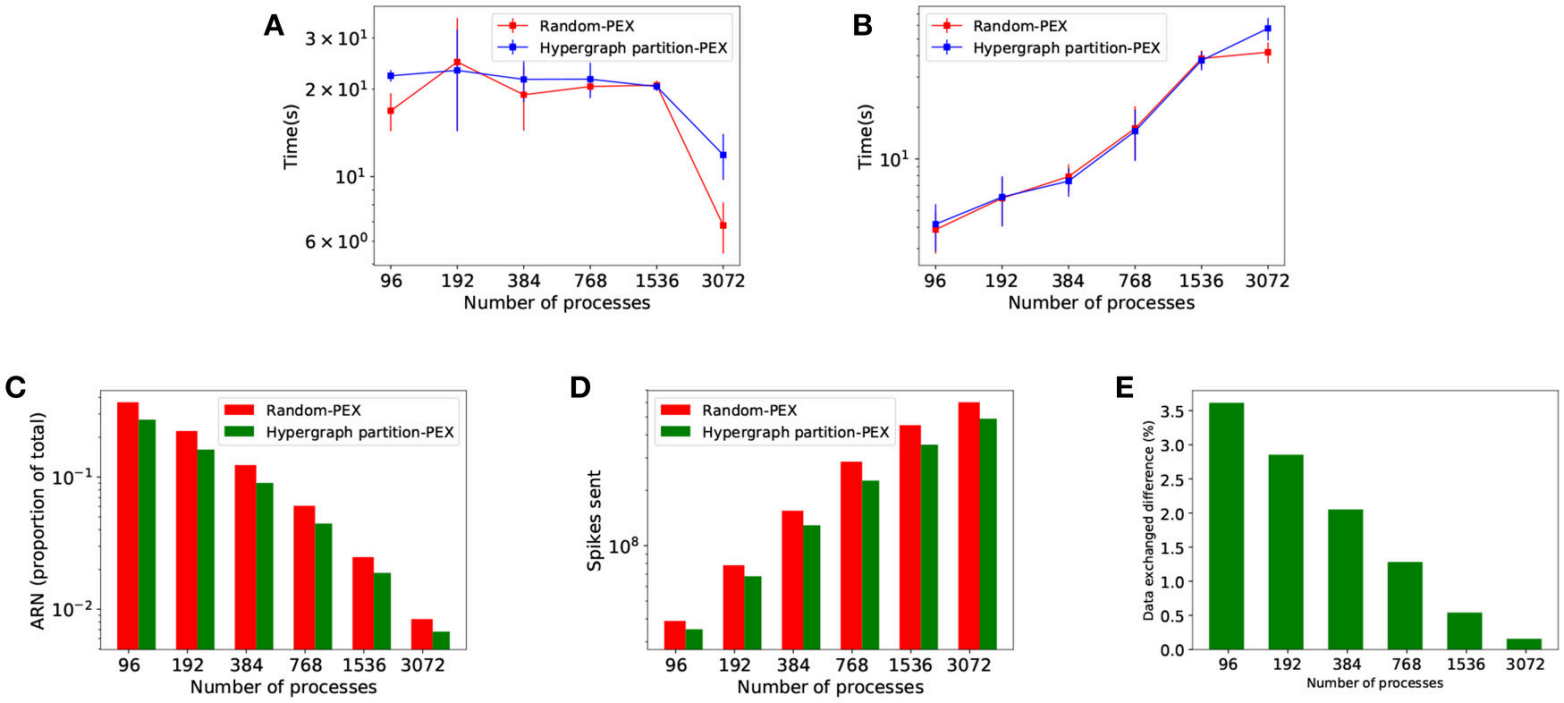
Performance results of *Random balanced* allocation compared with *hypergraph partitioning*. The top part shows quantitative timings during the different phases of communication: **(A)** Implicit synchronization time and **(B)** data exchange time. The bottom part of the figure shows reductions brought by hypergraph partitioning over random: **(C)** Average number of runtime neighbors (average number of processes each process communicates to at any given communication step, a subset of the total neighborhood defined in the PCG); **(D)** remote spikes (local spikes that need to be propagated to other processes); **(E)** spiking data volume exchanged difference.

Figure 7 shows comparative results from both allocation strategies. Since they are based on the same metrics for load balance, there are no differences in implicit synchronization time between strategies (Figure 7A). *Hypergraph* has its impact in terms of interprocess connectivity. As expected, the number of average runtime neighbors (ARN, average number of target processes at each communication step per process) is reduced (Figure 7C), making communication more sparse. This leads to fewer remote spikes, when a local neuron spike needs to be propagated to other processes (Figure 7D) and a decreased total amount of data exchanged (Figure 7E). Despite the improvement in sparsity, data exchange time (Figure 7B) is not significantly reduced and therefore simulation time is not affected since *PEX* is not designed to take advantage of sparsity—see discussion in section 4.1.

### 3.2. *NBX* Dynamic Sparse Communication Pattern

**Figure 8:**
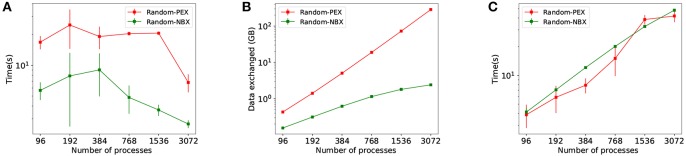
Communication time using *PEX* and *NBX* are shown. **(A)** Implicit synchronization time per simulation. **(B)** Total volume of spiking data exchanged during simulation. **(C)** Data exchange time per simulation.

*NBX* and *PEX* communication patterns are compared in simulations using *Random Balanced* allocation. Figure 8 shows how *NBX* reduces communication time by significantly decreasing implicit synchronization time (Figure 8A), as a result of a more balanced communication time amongst processes—see discussion in section 4.2. Total amount of spiking data sent across processes is decreased (Figure 8B) due to the elimination of the handshake phase during communication. Data exchange time is not impacted (Figure 8C) despite the reduction in data—see discussion in section 4.2.

### 3.3. Combining Neuron Allocation (HP) With and Sparse Communicator (*NBX*)

Once the strategies have been evaluated independently, we combine them to identify synergies. The four candidates compared are *Random-PEX, Random-NBX, Hypergraph partition-PEX*, and *Hypergraph partition-NBX*, indicating which neuron allocation algorithm (random balanced or hypergraph partitioning) and communication strategy (*PEX* or *NBX*) is used.

**Figure 9:**
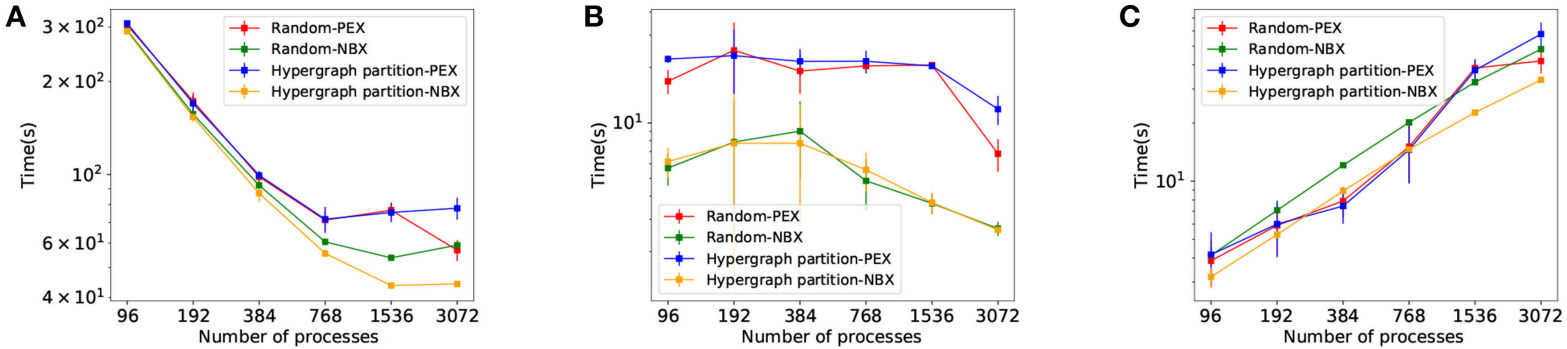
Simulation performance measurements for *Rand-PEX, HP-PEX, Rand-NBX*, and *HP-NBX*. **(A)** overall simulation time. **(B)** Implicit synchronization time per simulation. **(C)** Data exchange time per simulation.

The results in Figure 9 show the effectiveness of *Hypergraph partition-NBX* over the rest, with reduced simulation time (Figure 9A) as a consequence of decreased communication time. The improvement is due to implicit synchronization time gain (Figure 9B) brought by the use of *NBX*, as well as a reduction of data exchange time of *Hypergraph partition-NBX* over *Rand-NBX*—see discussion in section 4.3 for an in depth analysis.

The *Hypergraph partition-NBX* approach is not only faster than the random alternatives, but as Figure 9A demonstrates, it also manages to improve scaling by delaying the point at which the increased communication overhead outweighs the gains in simulation time. The approach scales up to 1,536 processes for this model (per 768 of the other alternatives).

### 3.4. Round Robin Allocation in Large Models

A second set of strong scaling experiments has been performed to analyse how *HP-NBX* scales to larger, modular models, such as the *MCV* model. The experiments involve simulating the MCV model over a 350 ms time interval with a 0.1 ms timestep, using the following range of scales: 192, 384, 768, 1,536, 3,072, and 6,144 processes.

Round robin scheduling of computations is a common workload distribution strategy in neuronal simulators. It is used as a simple load balancing approach, spreading neurons with similar activity patterns across the network (Jordan et al., 2018). Thus, round robin is used in here as a baseline, with *PEX* communication.

**Figure 10:**
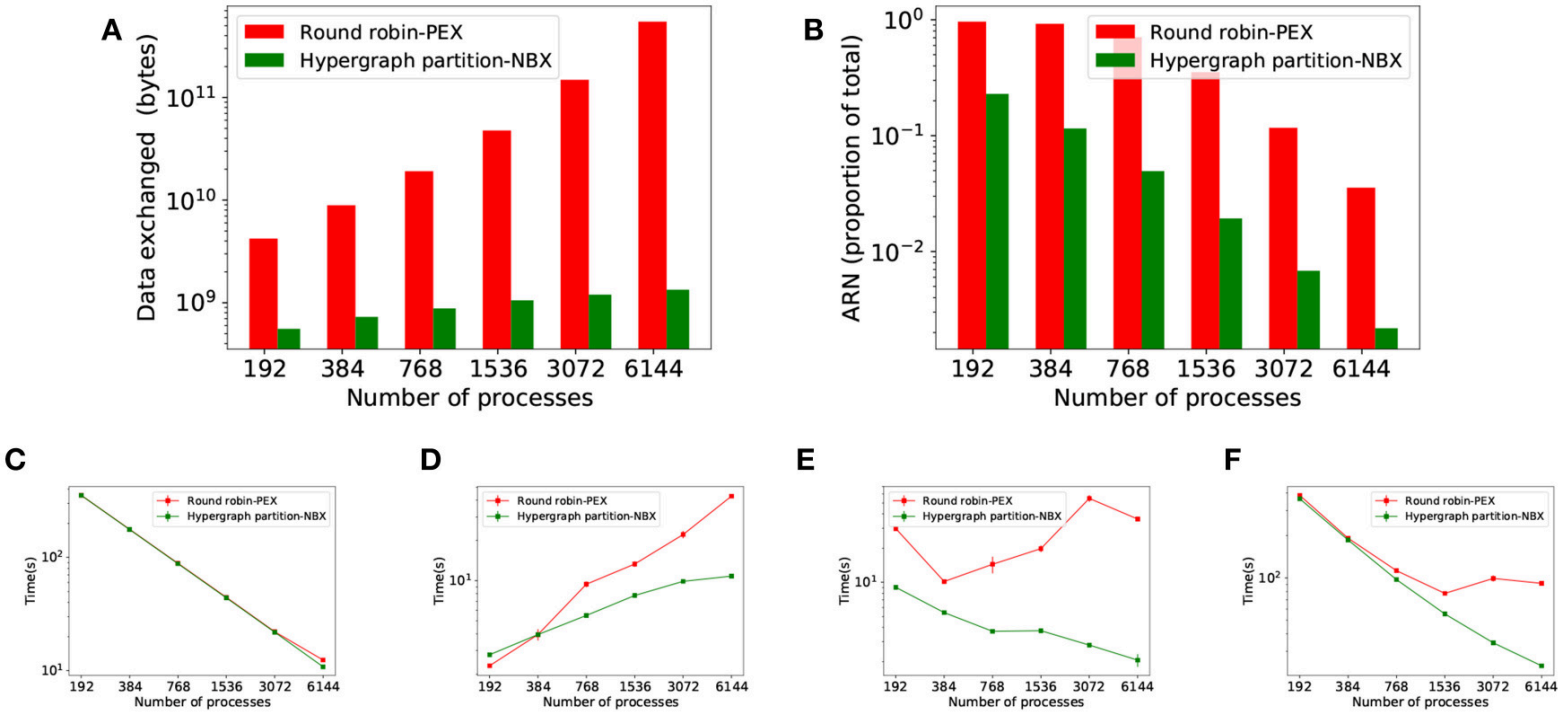
Simulation results of the *MVC* model showing performance improvements of *HB-NBX* over the baseline *round robin-PEX*. The top part of the figure displays connectivity-related metrics. **(A)** Data volume exchanged during simulation; **(B)** Average runtime neighbors in communication phases reduction in percentage. The bottom part of the figure shows results of the *MVC* scaling experiments with both alternatives. **(C)** Computation time which decreases linearly, demonstrating the potential benefit of increased parallelism; **(D)** Data exchange time; **(E)** Implicit synchronization time; **(F)** Total simulation time.

Figure 10 shows the results of the strong scaling experiments for the larger, modular MVC model. It compares our proposed strategy *HP-NBX* to the baseline *Round robin-PEX*. The computation time scales linearly with the number of processes (Figure 10C), indicating the simulation does benefit from parallel execution. *HP-NBX* significantly impacts interprocess communication by reducing the number of average runtime neighbors by around 90% (Figure 10B). Hosted neurons have to send spikes to fewer neighbors, and thus data exchanged is reduced in comparison (Figure 10A). This is a qualitative reduction, as the data volume has a quadratic growth for *Round robin-PEX* but linearly for *HP-NBX*. With less data volume being sent and a more balanced communication pattern, both phases of communication are reduced: data exchange time (Figure 10D) and implicit synchronization time (Figure 10E). As a consequence, *HP-NBX* simulation time scales well with the number of processes, whereas *Round robin-PEX* struggles with high process counts (no improvement with 1536 processes or higher).

**Figure 11:**
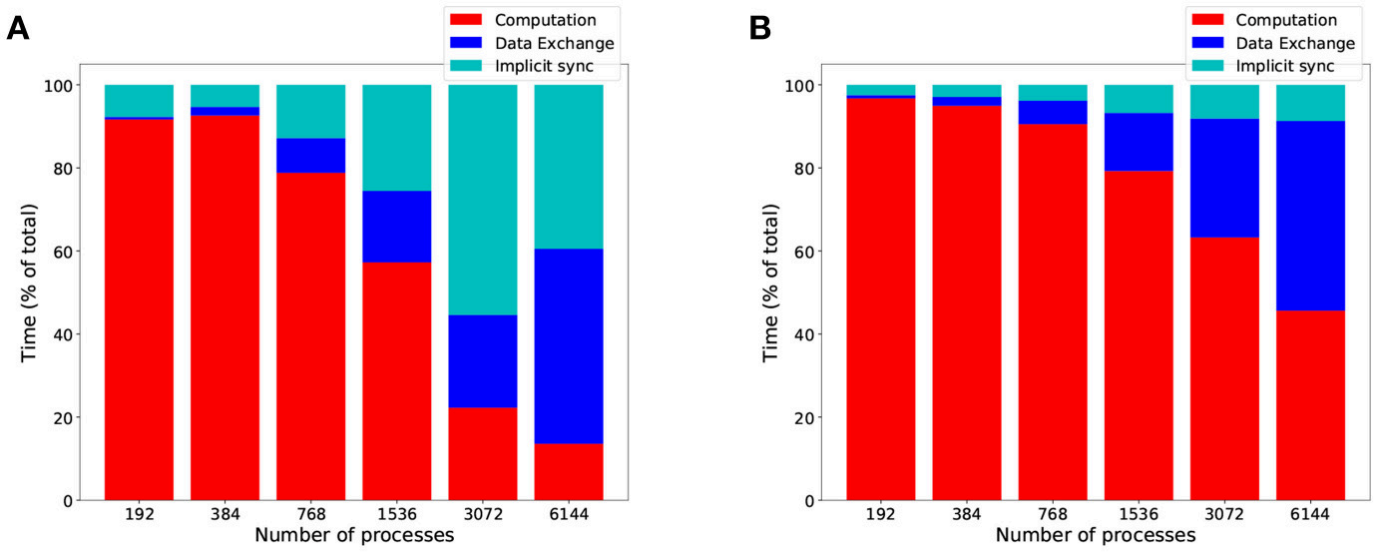
*HP-NBX* computational efficiency gains over the baseline *Round robin-PEX during MVC simulations*. The graph shows the proportion of time an MVC simulation spends in computation (red), implicit synchronization (light blue), and data exchange (dark blue) when using *Round robin-PEX* **(A)** and *HP-NBX* **(B)**. The proposed strategy *HP-NBX* consistently increases computational efficiency in all processes counts (percentage points improvement in increasing process count order: 5.2, 2.7, 12, 22.3, 40.8, and 32).

The overall computational efficiency gain of *HP-NBX* is outlined in Figure 11, which compares it to the baseline *Round robin-PEX*. Not only overall simulation time is reduced by up to 73% (Figure 10F) but the proportion of time spent in computation is increased (Figures 11A,B), resulting in an improved computational efficiency, by up to 40.8 percentage points (from 22.2 to 63%).

## 4. Discussion

Spike propagation, the necessary exchange of data between computational nodes, is shown to limit scalability in distributed SNN simulations—see Figure 1. Jordan et al. (2018) and Schenck et al. (2014) suggest that this exchange may not be limiting scalability for simulations that make effective use of message packing. Since such simulations employ synaptic delays that are larger than the resolution of the simulation time step, communication steps only occur on multiples of the global minimum synaptic delay. This significantly reduces the number of synchronization events and therefore minimizes the impact of communication on the overall simulation time. In contrast, for models that cannot make use of message packing (such as the *CM* and *MVC* models with random synaptic delays), communication is acknowledged as a bottleneck for scalability.

### 4.1. Hypergraph Partitioning as a Neuron Allocation Strategy

Our proposed neuron allocation method based on hypergraph partitioning offers an improvement to approaches employing graph partitioning (Heien et al., 2010) or clustering (Urgese et al., 2016). A hypergraph allows the total communication volume of the simulation to be modeled more accurately than using a normal graph (Devine et al., 2005; Deveci et al., 2015) which enables a better allocation of neurons to computing nodes to reduce connectivity between processes.

Hypergraph partitioning is effective in minimizing interprocess connectivity, as shown by a 10–20% reduction in remote spikes (Figure 7D) and 20–25% in average runtime neighbors (Figure 7C). Increased sparsity of the communication graph, however, does not impact data exchange time—see Figure 7B. This is because *PEX* is not designed to take advantage of this sparsity: send-receive data is implemented with MPI_Alltoall, which results in global synchronization of all processes even with reduced interconnectivity. Therefore, performance improvement on simulation time is negligible.

Partitioning alone brings a very moderate reduction in volume of data exchanged (Figure 7E) that becomes negligible as the parallelism is increased. The effect is expected since partitioning optimizes only the data send-receive portion of the exchange phase, and as shown in Figure 5 this is not the dominant part of the communication, less so as the number of processes increases. Parallel SNN simulations in which the send-receive part is dominant (e.g., high frequency of neuronal activity or increased neuronal density per process) could see a significant improvement here.

Lytton et al. (2016), point out that simple collective communication patterns like *all gather* may perform better than point-to-point provided the number of ranks is significantly lower than the average number of connections per neuron. This tipping point assumes random allocation of neurons and hence random chance of post-synaptic targets being placed at any computing node. Using partitioning actively increases the probability of a pre- and post-synaptic neuron living in the same process (as seen by a reduction in runtime neighbors in Figure 7C) and therefore neurons will normally have less targeted process than post-synaptic targets. This shifts the tipping point in favor of point-to-point communications to be reached much sooner, at fewer processes.

### 4.2. *NBX* Dynamic Sparse Exchange

*NBX* has a strong impact on implicit synchronization time (Figure 8A), which is an indirect measurement of load balance between processes. With both alternatives employing *Random Balanced* allocation, the computation phase remains the same. Therefore any change in load balance, reflected in implicit synchronization, can be attributed to the communication strategy—some processes taking longer than others to finish communication phase which carries over to the next communication step when processes re-synchronize again. *PEX* makes use of collective MPI calls, which ensure that processes are synchronized at the point of entry of that function. Note that each process may continue execution as soon as it has receive messages from all others. This is not guaranteed to happen at the same time for all processes, introducing imbalance—a well-known phenomena in parallel synchronization (Hoefler et al., 2010a). With *NBX* processes are implicitly synchronized with an asynchronous MPI_Ibarrier: all processes are guaranteed to have sent all data before any of the processes finishes communication phase and continues with the simulation. This acts as a balancing mechanism whilst not forcing processes to be idle, since the barrier notification messaging happens in parallel whilst receiving data.

The cost of synchronization for *NBX* shows in the measured time for data exchange on Figure 8C. *NBX* scales in a more predictable way than *PEX*, but at the level of communication of the simulation (random allocation of neurons leads to almost all to all process connectivity) it is often slower. Hoefler et al. (2010b) indicates that the performance of *NBX* is dependent on the number of neighbors (sparsity) during communication. Hence, increasing sparsity would improve *NBX* performance—see discussion in section 4.3.

Figure 8B shows volume of data sent by both alternatives, with a qualitatively improvement of *NBX*. This measurement accounts only for explicit data sent; for *PEX* this includes handshake metadata, which as we have discussed becomes dominant with increased parallelism (each process needs to send data to all other processes specifying whether further communication is to be expected). *NBX* performs the handshake implicitly with MPI_Ibarriers, which has a payload of 0 and does not require all to all messaging.

### 4.3. Synergy Between Hypergraph Partitioning and *NBX*

The combined *Hypergraph partition-NBX* strategy performs better than either one on its own (Figure 9A). It keeps the reduced implicit synchronization time (Figure 9B) that comes with *NBX* better process balance during communication. Furthermore, when compared to *Random-NBX, Hypergraph partition-NBX* has enhanced communication sparsity due to partitioning allocation that increases the effectiveness of the sparse communication pattern, resulting in data exchange time reduction (Figure 9C). With sparsity, *Hypergraph partition-NBX* matches the data exchange performance of *PEX* alternatives in lower processor counts, and improves upon it as the parallelization increases.

The dual nature of *Hypergraph partition-NBX* could impact performance in SNN models with different communication profiles. In high frequency communicating SNN simulations (high neuron firing rate or high process neuronal density), where communication is dominated by data send-receive (Figure 5), the impact of hypergraph partitioning in reducing the volume of communication is expected to increase: when more neurons are spiking, more data is sent across processes, making any reduction in inter-process synapses more significant. This effectively increases the sparsity of communication, improving the effectiveness of *NBX*. In low frequency communicating SNN simulations (low neuron firing rate, low process neuronal density, or a modular SNN model that can be partitioned well), where communication is sparse, *NBX* is expected to speed up data exchange time—as discussed in section 4.2 and by Hoefler et al. (2010b). Therefore, with increased network communication sparsity, *NBX* is a more suitable communication pattern than point-to-point strategies used by SNN distributed simulators (Hammarlund and Ekeberg, 1998; Kumar et al., 2010; Minkovich et al., 2014; Jordan et al., 2018).

### 4.4. *HP-NBX* vs. *Round Robin* in Large and Modular Model

We have shown that the proposed strategy leads to significant performance improvement on the *CM* model. The main limitations of this model are its relatively small size and the random connectivity between its layers, which can pose a challenge to workload distribution (particularly the random connectivity, as it makes reducing communication through partitioning more challenging). To demonstrate further applicability of our strategy to larger and modular models, we investigate the effect of *HP-NBX* on the *MVC* model: 660 k neurons with 620 million synapses, arranged in 32 modular areas as described by Schmidt et al. (2018).

Round robin is a standard neuron allocation algorithm employed by many neuronal simulators (such as NEST or NEURON). Although it is an adequate load balancing approach (Jordan et al., 2018), round robin represents the worse-case scenario in terms of connectivity, as it purposefully separates neurons which are more likely to be more interconnected (those belonging to the same population). As a consequence, round robin forces each process to be communicating with a high number of other processes.

*HP-NBX* is capable of taking advantage of the modularity of the model and decreases the average number of neighbors each process communicates with by 90% (Figure 10B). The lower ARN impacts the data exchanged not only in reducing its volume but also allowing linear scaling with the number of processes, compared to the quadratic growth of *Round robin-PEX*.

The decreased ARN allows the *NBX* algorithm to be more efficient and balanced and hence simulations spend less time in implicit synchronization (Figure 10E). The qualitative difference in scaling of data volume (linear for *HP-NBX* and quadratic for *Round robin-PEX*) leads to reduced data exchange time (Figure 10D). Both Figures 10D,E show how our approach scales better: data exchange time growth slows down with the number of processes, in contrast with a continuous quadratic growth of the baseline; similarly, implicit synchronization time decreases with higher processor counts, whereas it is increased in the baseline. This limits the scalability of simulations with *Round robin-PEX* (Figure 10F), with simulation time not being improved despite the extra computing resources (1,536 processes and above). In contrast, *HP-NBX* scales well, with simulation time reduced for all process counts.

The impact of *HP-NBX* on data exchange time reduction contrasts with those seen when comparing random allocation and hypergraph partitioning using the *CM* model, in which a moderate reduction on average runtime neighbors (25%) or data volume (1–3%) on their own did not significantly impact data exchange time. There are two key factors that explain the difference. The first one is quantitative: the communication reduction is much greatermore intense in the *MVC* model (90% average runtime neighbors and 80% data volume). The second reason is qualitative and is due to the scaling of both data volume and ARN. The data volume difference (Figure 7E) and the ARN difference (Figure 7C) in the CM simulation decreases with the number of processes, whereas both differences increase rapidly in the *MVC* model, making it more significant the more the simulation is parallelized. This is therefore sufficient to impact communication time.

Overall Figure 10 shows that *HP-NBX* is a more effective allocation and communication algorithm than *Round robin-PEX*, yielding shorter simulation times (Figure 10F). It is worth noting that our implementation of round robin does not take advantage of short cuts such as finding the target process for a post-synaptic neuron by simply using mod operator, however this is a memory optimization rather than a computation one and therefore does not affect our results. This results demonstrate the need of careful neuron allocation based on connectivity and the inadequacy of round robin on large scale communication bound simulations.

### 4.5. Cost of *HP-NBX* Against the Gains in Simulation Time

**Figure 12:**
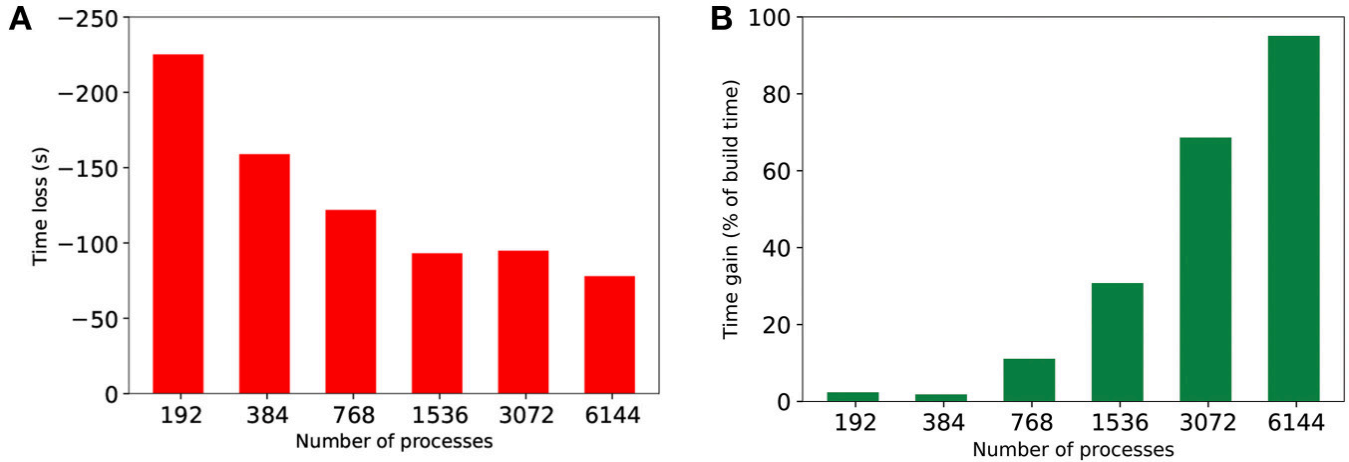
Comparing the cost of computing hypergraph partitioning in extra build time (in seconds) **(A)** to the gains in simulation time by *HP-NBX* as a percentage of the build time **(B)**. Notwithstanding the gains on a 350 ms simulation are canceled out by the extra computing cost, the gains are expected to scale with the simulated time, whereas the cost is uniform for any given simulation time chosen and number of processes.

Performing hypergraph partitioning adds a cost to building the simulation that is calculated by taking the difference between build time for baseline and *HP-NBX*. Similarly, the time gain of the simulation is defined as the difference in simulation time between the baseline and *HP-NBX*, as a percentage of the build time. Figure 12 shows the build time and simulation time gain between *Round robin-PEX* and *HP-NBX* in 350 ms of MVC simulations. Due to the use of a parallel implementation of hypergraph partitioning, build time difference (Figure 12A) is reduced when scaling. With lower simulation time (Figure 10F), the time gain of *HP-NBX* reaches over 90% with 6,144 processes. Although this means the proposed strategy is under 10% slower overall (build time and simulation time), this is the case for a short simulation of 350ms. Simulation time gains are proportional to the simulated time but build time is independent of it. Therefore, as the simulated time increases, simulation gains will continue to increase whilst the build time remains the same, resulting in the partitioning cost becoming negligible.

## 5. Conclusions

Communication becomes the dominant part of parallel SNN simulations as the number of processes is increased, limiting scalability. This work shows how to improve computational efficiency in distributed SNN simulations by optimizing the three phases of communication: implicit synchronization, process handshake, and data exchange. We use neuronal connectivity to reduce interprocess communication, applying hypergraph partitioning to drive neuron allocation to processes. To tackle the impact of the mandatory handshake in point-to-point parallel communications, we implement *NBX* dynamic sparse strategy as a communication pattern.

This work demonstrates:

1. Hypergraph partitioning leads to communication sparsity in distributed SNN simulations and reduces volume of data exchanged.
2. Dynamic sparse communication *NBX* smooths process load imbalance introduced by *PEX*, resulting in reduced implicit synchronization time.
3. Synergy between partitioning and *NBX*: Hypergraph partitioning sparsity makes *NBX* more effective and reduces data exchange time.
4. Hypergraph partitioning neuron allocation combined with *NBX* communication pattern increases computational efficiency by up to 40.8% points and reduces simulation time by up to 73%, compared to round robin allocation, a standard algorithm in neuronal simulators.

The findings are application agnostic and are applicable to other distributed complex system simulations in which communication can be modeled as a graph network.

### 5.1. Future Work

This work has considered only uniform network topology of processes, i.e., the cost of communication between any two processes remains equal. Given the heterogeneity in High Performance Computing, where compute nodes are located at increasing distances (sharing a board, within the same network group, or at remote groups), considering the physical topology of the distributed computing network could have an impact on neuronal allocation. This information can be used to weigh the PCG to better estimate the cost of communication between processes.

Although the communication scalability problem has not been removed completely (Figure 11), it has been reduced and pushed to the right (i.e., to a higher compute node count). Implicit synchronization due to load imbalance in communications has become negligible and constant when scaling. The data exchange phase of communication remains a target to optimize for future work.

Our study is focused on constant current injection to generate reproducible activity patterns across processes. The impact of biologically plausible activity patterns of spiking neurons remains an interesting extension of this work.

## Author Contributions

All authors contributed to the conception and design of the work. CF-M wrote the first draft of the manuscript. PR and DC performed substantial critical revisions and contributed to the experimental design. CF-M implemented the code. All authors contributed to manuscript revision, read and approved the submitted version.

### Conflict of Interest Statement

The authors declare that the research was conducted in the absence of any commercial or financial relationships that could be construed as a potential conflict of interest.

## Footnotes

^1^The details of the 77 k neurons model are described in section 2.3.1.

^2^This is implemented with MPI_Barrier calls. Although there are limitations to this approach (a process is only held until it is notified that all other processes have entered the barrier; but message propagation delays may result in additional imbalance) it is a good approximation of time to synchronize.

^3^More information regarding ARCHER hardware can be found on https://www.archer.ac.uk/about-archer/hardware/

^4^This value is the default time step in many SNN simulators as a resolution for the simulation that provides meaningful functional results.
